# Assessing the Rheological, Mechanical, and Photocatalytic Properties of Niobium Oxide-Incorporated White Cement Pastes

**DOI:** 10.3390/ma16114090

**Published:** 2023-05-31

**Authors:** Laura Silvestro, Caroline Maroli, Brenda Koch, Artur Spat Ruviaro, Geannina Lima, Mariane Kempka, Camila Fabiano de Freitas Marin, Daniela Zambelli Mezalira, Philippe Jean Paul Gleize

**Affiliations:** 1 Coordenação de Engenharia Civil, Universidade Tecnológica Federal do Paraná (UTFPR), Guarapuava 85053-525, Brazil; marianekempka@professores.utfpr.edu.br; 2 Programa de Pós-Graduação em Química, Universidade Federal de Santa Catarina (UFSC), Florianopolis 88040-900, Brazil; c.maroli@posgrad.ufsc.br (C.M.); bbrendakoch@gmail.com (B.K.); camila.f.freitas@ufsc.br (C.F.d.F.M.); daniela.z.m@ufsc.br (D.Z.M.); 3 Laboratório de Aplicação de Nanotecnologia em Construção Civil (NANOTEC), Universidade Federal de Santa Catarina (UFSC), Florianopolis 88040-900, Brazil; arturspatruviaro@gmail.com (A.S.R.); geanninasantos@hotmail.com (G.L.); p.gleize@ufsc.br (P.J.P.G.)

**Keywords:** niobium, cement, photocatalytic, rheology, hydration

## Abstract

Niobium oxide (Nb_2_O_5_) is a semiconductor that exhibits photocatalytic properties, making it potentially valuable in addressing air pollution, self-cleaning, and self-disinfection in cement-based materials (CBMs). Therefore, this study aimed to evaluate the impact of different Nb_2_O_5_ concentrations on various parameters, including rheological characteristics, hydration kinetics (measured using isothermal calorimetry), compressive strength, and photocatalytic activity, specifically in the degradation of Rhodamine B (RhB) in white Portland cement pastes. The incorporation of Nb_2_O_5_ increased the yield stress and viscosity of the pastes by up to 88.9% and 33.5%, respectively, primarily due to the larger specific surface area (SSA) provided by Nb_2_O_5_. However, this addition did not significantly affect the hydration kinetics or the compressive strength of the cement pastes after 3 and 28 days. Tests focusing on the degradation of RhB in the cement pastes revealed that the inclusion of 2.0 wt.% of Nb_2_O_5_ was insufficient to degrade the dye when exposed to 393 nm UV light. However, an interesting observation was made concerning RhB in the presence of CBMs, as it demonstrated a degradation mechanism that was not dependent on light. This phenomenon was attributed to the production of superoxide anion radicals resulting from the interaction between the alkaline medium and hydrogen peroxide.

## 1. Introduction

The utilization of nanomaterials in cement-based matrices is a promising alternative for creating composites suitable for specific applications. The literature extensively documents the reinforcing capability of nanomaterials in enhancing the mechanical properties of cement-based materials (CBMs) [1,2,3,4,5,6,7,8]. Furthermore, titanium dioxide (TiO_2_) nanoparticles offer photocatalytic activity to CBMs, allowing for applications such as air pollution remediation, self-cleaning, and self-disinfection [9]. Previous studies have reported the ability of TiO_2_ cement-based materials to degrade pollutants such as sulfur dioxide (SO_2_) [10] and NO_x_ gases [11,12] and inhibit algae growth [13]. Therefore, cementitious composites containing TiO_2_ contribute to the maintenance and service life of buildings, leading to reduced cleaning costs. Moreover, they have the potential to serve as materials for mitigating urban air pollution [9,14].

Photocatalysis involves the utilization of UV light (with a wavelength <400 nm) to activate TiO_2_ nanoparticles, which decompose organic compounds [15,16]. As a result, cementitious materials containing TiO_2_ are commonly applied to external building surfaces or pavement concretes. These applications possess a large surface area, leading to increased exposure to sunlight and, consequently, enhanced photocatalytic activity [9]. Another material with potential photocatalytic properties is niobium oxide. Kumari et al. [17] investigated different phases of Nb-oxide and identified its photocatalytic activity through UV and visible radiation for the degradation of aqueous probe pollutants. The authors suggested that pentoxide niobium could be a promising alternative to current photoactive nanomaterials due to its suitable band gap, band edge location, stability, sorption properties, and recyclability. Chebanenko et al. [18] observed that rhombic niobium pentoxide (Nb_2_O_5_) exhibited the highest photocatalytic activity for the oxidation of an aqueous solution of methyl orange when exposed to visible light. Similarly, Ücker et al. [19] demonstrated the potential of Nb_2_O_5_ in degrading rhodamine B (RhB), suggesting its use for removing organic pollutants from effluents. Tamai et al. [20] synthesized Nb_2_O_5_ nanoparticles for the selective photooxidation of benzyl alcohol and found that the photocatalytic activity correlated with the surface area of Nb_2_O_5_—the higher surface area associated with greater catalytic activity.

However, there has been limited exploration of the photocatalytic properties of niobium oxide as an alternative to TiO_2_ in CBM applications. Interestingly, research on niobium (Nb) is of particular relevance to Brazil, as the country possesses the world’s largest reserves of niobium, accounting for approximately 98.5% of the global total. Additionally, Brazil is the largest producer of Nb-metal, contributing to around 97.2% of global production [21]. One of the few studies on this topic is by Moreira et al. [22]. The authors assessed the photocatalytic activity of Portland cement pastes containing Nb_2_O_5_, which was activated using visible and ultraviolet (UV) light. The addition of Nb_2_O_5_ modified the bandgap associated with the photocatalytic activity of cement-based materials, resulting in reduced degradation of methylene blue dye. These results may be attributed to the micrometric size of the Nb_2_O_5_ particles, with an average diameter of 11.4 µm [22]. Consequently, the effect of nanometric Nb_2_O_5_ particles on the properties of cementitious matrices remains unexplored.

There is a scarcity of studies focusing on the incorporation of Nb_2_O_5_ in CBMs. This lack of research makes it challenging to determine the appropriate Nb_2_O_5_ content and dispersion method for cementitious matrices. To address this, studies investigating the application of TiO_2_ were examined to identify relevant parameters, as TiO_2_ shares similar characteristics with Nb_2_O_5._ Table 1 summarizes selected studies on the topic. Typically, TiO_2_ contents ranging from 2 to 3% were employed, with some studies utilizing higher values up to 10 wt.%. In addition, the dispersion of nanomaterials is typically achieved using sonication as the preferred method. Regarding the sonicator setup, it can be observed that duration times up to 30 min are applied. Reches et al. [23] also noted that over 95% of the TiO_2_ deagglomeration could be achieved within 20 min.

The study aims to evaluate the effect of incorporating Nb_2_O_5_ nanoparticles on the properties in the fresh and hardened state and the photocatalytic activity of white Portland cement pastes. To evaluate the rheological properties, rotational rheometry tests were conducted during the initial two hours of cement paste hydration. In addition, hydration kinetics were evaluated using isothermal calorimetry, and compressive strength was determined after 3 and 28 days. The photocatalysis tests assessed the capacity of cement pastes containing 2% Nb_2_O_5_ to degrade organic compounds, specifically RhB. This test serves as a means to evaluate the self-cleaning properties of CBMs.

## 2. Materials and Methods

### 2.1. Materials

**Figure 1:**
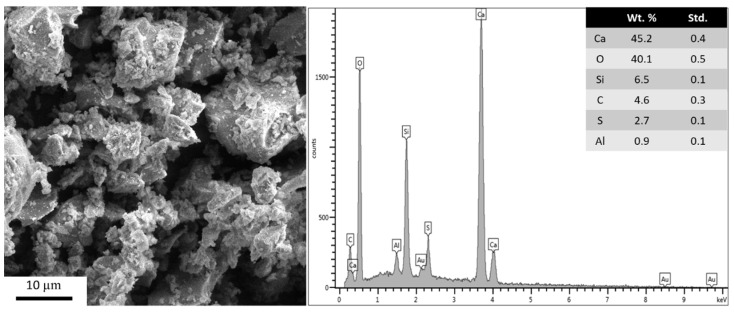
SEM image and EDS of white Portland cement [×5000 magnification].

A white Portland cement (WC) was utilized to produce the cement pastes analyzed in this study. The chemical and physical properties of WC are presented in Table 2. The chemical composition was determined using X-ray fluorescence (XRF) with a ZSX Primus IV (Rigaku) spectrometer. The particle size distribution of the cement was measured using an S3500 Particle Size Analyzer (Microtrac). The specific surface area (SSA) was determined using the Brunauer–Emmett–Teller (BET) method, employing nitrogen adsorption using a Nova Station A (Quantachrome). Prior to analysis, degasification was performed at 300 °C for 1 h. Figure 1 shows the scanning electron microscopy (SEM) [35] image of WC and its composition determined by energy dispersive spectroscopy (EDS). SEM images were obtained using a VEGA3 (TESCAN) microscope operating at 15 kV, while EDS analysis was also conducted on this sample using an X-art (Oxford) detector. WC is mainly composed of calcium (Ca), oxygen (O), and silicon (Si). Notably, the WC composition exhibits a low iron (Fe) content, which is responsible for its white color.

**Figure 2:**
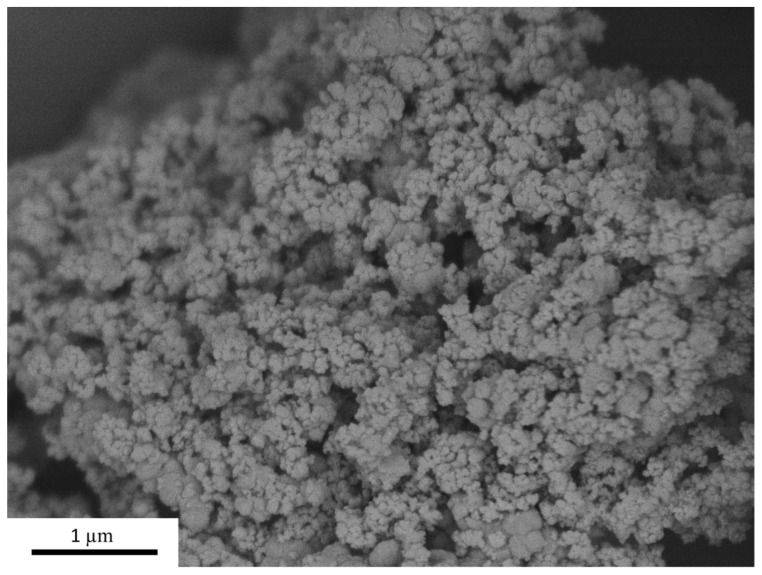
SEM image of Nb_2_O_5_ [×20,000 magnification].

A commercial Niobia HY-340 is a niobium oxide hydrate with an approximate Nb_2_O_5_ content of 80.0%, obtained from Companhia Brasileira de Metalurgia e Mineração (CBMM). The specific surface area (SSA) of the nanomaterial was determined to be 135 m^2^/g using the same equipment and conditions mentioned earlier. The SEM image of Nb_2_O_5_ in Figure 2 shows its tendency to agglomerate, with individual nanoparticles exhibiting an average diameter of approximately 100 nm. This image also highlights the need to employ a dispersion process, such as sonication, to ensure proper dispersion of the nanoparticles.

### 2.2. Mixture Design

The cement pastes in Table 3 were prepared with a water-to-binder (w/b) ratio of 0.4. Various Nb_2_O_5_ addition contents, ranging from 0.5 to 2.0% by the weight of cement, were assessed. To isolate the effect of Nb_2_O_5_ nanoparticles, no superplasticizer admixture was used. Initially, the Nb_2_O_5_ contents were added to the water and dispersed using a probe sonication Vibra–Cell with a diameter of 13 mm (VCX Serie, 750 W, 20 kHz). The sonication process was carried out at an amplitude of 50% for 30 min. To prevent overheating of the Nb_2_O_5_ aqueous dispersions, the sonication was performed in an ice bath. The dispersions were added to the cement and mixed manually for 30 s. After 20 s of rest, the mixture was mechanically mixed for 70 s using a high-shear mixer (10,000 rpm). The Nb_2_O_5_ contents and the sonication parameters were defined based on the studies presented in Table 1.

### 2.3. Experimental Methods

Rotational rheometry tests were conducted on white cement pastes using a Haake MARS III (Thermo Fisher Scientific, Waltham, MA, USA) rheometer at a temperature of 23.0 °C. A hatched parallel-plate geometry with a diameter of 35.00 mm and an axial gap of 1.000 mm was used. The measurements started 10 min after the contact of WC with water and were conducted at 10 min intervals for a duration of 2 h, resulting in 12 measurements over this period. To prevent water evaporation, an insulation chamber was employed. The test routine started with a pre-shear at a shear rate of 100 s^−1^ applied for 60 s. The ascending flow curve was determined by increasing the shear rate from 0.1 to 100 s^−1^ in 10 steps. The descending flow curve was obtained by decreasing the shear rate from 100 to 0.1 s^−1^ in the same steps. In each measurement point, the shear rate was kept for 10 s to ensure shear stress stabilization and only the last 3 s of each stage were recorded. Data from the descending flow curve was fitted using the Herschel–Bulkley (H–B) model, as described in Equation (1). The equivalent plastic viscosity of cement pastes was calculated using Equation (2) [36].

(1) $$\tau =\tau_{0}+K\cdot {\dot{ɣ}}^{n}$$

(2) $${µ}_{eq}=\frac{3K}{n+2}\cdot {\left({\dot{ɣ}}_{max}\right)}^{n-1}$$

where τ is the shear stress (Pa), $\tau_{0}$ is the dynamic yield stress (Pa), $\dot{ɣ}$ is the shear rate (s^−1^), *K* and n are, respectively, the consistency and the pseudoplastic parameters of the H–B model, and ${\dot{ɣ}}_{max}$ is the maximum shear rate applied.

For the isothermal calorimetry test, approximately 10 g of the cement pastes were added to the vials after the mixing procedure described in Section 2.2. The heat released was measured at 23 °C for 72 h using a TAM Air (TA Instruments) calorimeter. The heat flow and cumulative heat results were normalized per gram of cement weight. The compressive strength of pastes was evaluated at 3 and 28 days following ASTM C1231 [37]. Five cylindrical specimens (Ø20 mm × h 26 mm) were cast for each cement paste.

**Figure 3:**
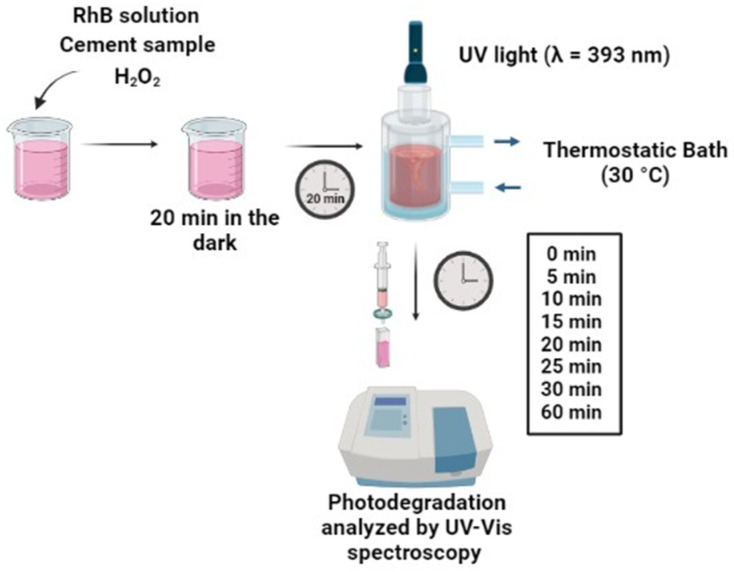
Schematic illustration of the photocatalytic degradation setup.

The photocatalytic activity of 0.0% Nb and 2.0% Nb cement pastes was analyzed by degrading the RhB dye [C_28_H_31_CIN_2_O_3_] in an aqueous solution with a concentration of 10 mg L^−1^. The test was conducted in a jacketed glass reactor (200 mL capacity) irradiated by a LED CREE UV lamp JYX-8860 with a maximum wavelength of 393 nm positioned 5 cm above the solution. Previous studies on the degradation of RhB with the Nb_2_O_5_ also utilized lamps with a similar wavelength (<380 nm) [38,39]. Initially, the cement samples were manually ground and sieved in a 75 µm opening mash. Subsequently, 0.1 g of the samples were added to the 100 mL of RhB solution and kept for 20 min in the dark to stabilize the adsorption–desorption equilibrium, similar to the approach adopted by Ücker et al. [19]. After 20 min of stabilization, the lamp was turned on, and a 3.0 mL aliquot of the solution was collected after 0, 5, 10, 15, 20, 25, 30, and 60 min, filtered with a PTFE syringe filter (0.22 µm), and analyzed by UV–Vis spectrophotometry (USB4000, Ocean Optics, Orlando, FL, USA). A representative scheme of the test setup is presented in Figure 3. The degradation at predetermined intervals was monitored by the UV absorption spectra characteristic peak of RhB at 554 nm [38,40]. All analyses of RhB degradation were conducted with the addition of hydrogen peroxide (1 mL) as an oxidizing agent and at a controlled reaction temperature of 30 °C. RhB photolysis and Nb_2_O_5_ (0.1 g) photocatalysis under the same experimental conditions was carried out. In addition, images were recorded to track the dye degradation of the 0.0% Nb and 2.0% Nb samples over time (0 and 24 h). The pH of the samples was checked both at the beginning and after 24 h.

The efficiency degradation is defined as [41]:

(3) $$Degradation\left(\%\right)=\frac{C_{0}-C_{t}}{C_{0}}\times 100$$

where C_0_ is the initial concentration of Rhodamine B (mg L^−1^), and C_t_ is the concentration of RhB at the time t (mg L^−1^). Since absorbance is directly proportional to dye solution concentration, organic dye solution absorbance values are used to quantify photodegradation. The concentration of RhB dye was calculated using the calibration curve.

## 3. Results

### 3.1. Rotational Rheometry

**Figure 4:**
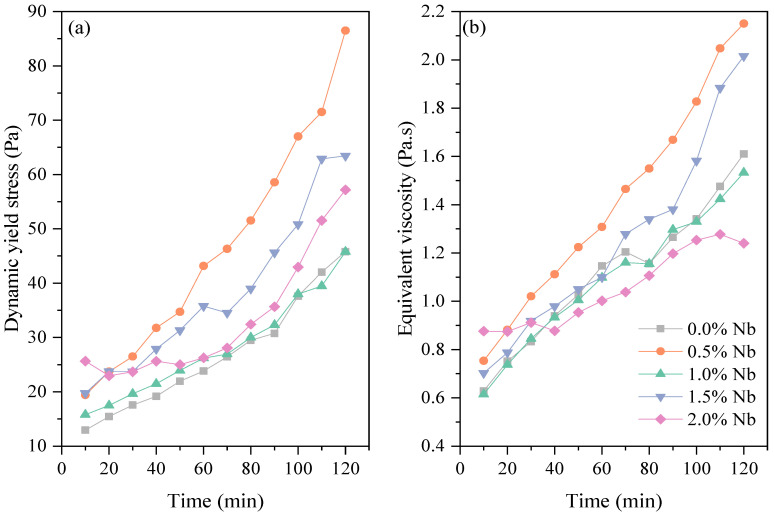
Rheological parameters of Nb cement pastes over 2 h: (**a**) dynamic yield stress, (**b**) equivalent viscosity.

For all evaluated compositions, the dynamic yield stress and equivalent viscosity of WC pastes increased over time, as shown in Figure 4. WC is characterized by higher tricalcium aluminate (C_3_A) contents compared to conventional Portland cement. As a result, it usually presents rapid loss of fluidity, reduced induction period, and shorter setting time [42]. In fact, the white cement pastes evaluated in this study showed significant increases in both rheological parameters within the first 60 min of hydration. After 60 min, there were observed increments of 84.0% and 82.3% in dynamic yield stress and viscosity, respectively, compared to the initial measurement at 10 min for the 0.0% Nb cement paste. These increments were 253.7% and 159.7% after 120 min of hydration for the plain cement paste (0.0% Nb).

In the evaluation of time-resolved yield stress for grey Portland cement (PC), Liu et al. [43] observed that a cement paste with polycarboxylate superplasticizer (SP) exhibited nearly constant yield stress during the first 30 min. However, between 30 and 180 min, the yield stress showed a linear increase, reaching a value approximately 60.0% higher than the initial measurement. Similarly, Silvestro et al. [44] observed a 169% increase in the dynamic yield stress from 10 to 60 min, an increase for a grey Portland cement paste with a w/c of 0.4 and a superplasticizer content of 0.2 wt.%. This progressive increase in the rheological parameters with increasing hydration time is expected and can be attributed to system flocculation and the formation of hydration products, which results in the formation of a rigid network [45].

Furthermore, the rapid formation of ettringite can also account for the increase in rheological parameters [46]. Therefore, this is of great significance, particularly in the case of white Portland cement matrices, which have a high C_3_A content. The substantial water incorporation within the ettringite structure promotes an increase in the rheological parameters of CBMs [46,47].

Regarding the effect of Nb_2_O_5_ incorporation on the rheological parameters of white cement pastes, it was observed that the progressive increase in dynamic yield stress and equivalent viscosity occurred after 10 min of hydration, which can be attributed to the smaller particle size and higher specific surface area (SSA) of niobium. A similar trend was observed for the other evaluated times. For instance, after 120 min of hydration, increases of 88.9% (0.5% Nb), 38.5% (1.5% Nb), and 24.8% (2.0% Nb) in the dynamic yield stress were observed compared to the plain cement paste. This effect became more pronounced at longer hydration times, possibly due to the additional surface area provided by niobium nanoparticles, facilitating the nucleation and growth of hydrated products [48,49]. Similar results were reported for the incorporation of other nanomaterials in CBMs, such as carbon nanotubes [44,50], titanium dioxide [49,51], and nano-silica [52,53,54].

Additionally, rotational rheometry results suggest that there exists an optimal Nb_2_O_5_ content for incorporation into cement pastes. Note that a 2.0% Nb content exhibited yield stress equivalent to the reference paste. However, the equivalent viscosity values were lower than those of the control sample for hydration times exceeding 90 min. This observation may suggest that this particular Nb_2_O_5_ content could have influenced the initial hydration reactions of the cement. As viscosity is linked to the flow of water within the porosity of the granular system, it is also associated with suspension concentration [55]. Hence, it is possible that a 2.0 wt.% Nb_2_O_5_ content slightly delayed the initial hydration reactions of white cement (within 120 min), resulting in viscosity values up to 23.0% lower than those of the plain cement paste. To gain a deeper understanding of the effect of niobium nanoparticles on the cement hydration process, a more comprehensive analysis, such as utilizing the in situ XRD technique, should be conducted.

### 3.2. Isothermal Calorimetry

**Figure 5:**
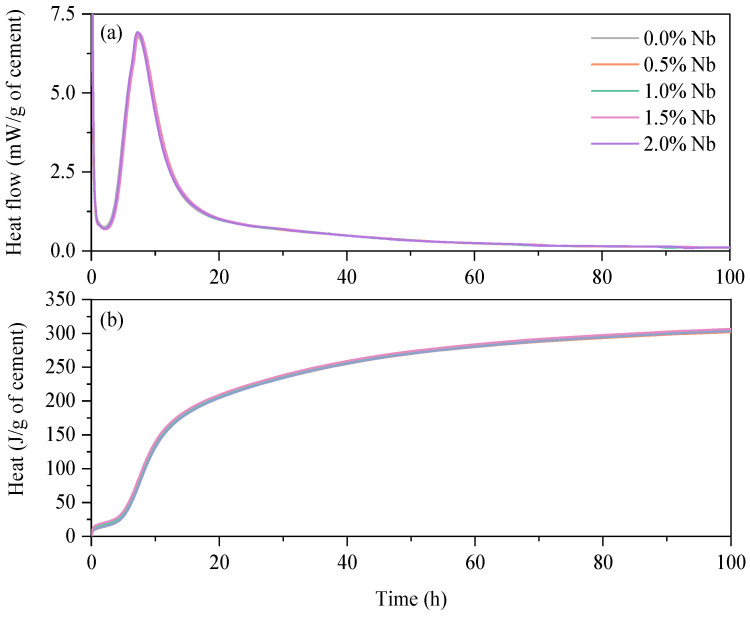
Heat flow (**a**) and Cumulative heat (**b**) of cement pastes containing different Nb_2_O_5_ contents.

The four main stages of the Portland cement hydration process can be identified in Figure 5, namely: (i) initial, (ii) induction, (iii) acceleration, and (iv) deceleration periods [56]. The main heat flow peak of WC pastes occurred at approximately 7.3 h. Note that this induction period is typically shorter compared to grey cement pastes, as indicated by the results reported by Scolaro et al. [57]. The authors observed that the main heat flow peak for a grey PC paste with a water-to-cement ratio (w/c) of 0.45 and a superplasticizer (SP) content of 0.025 wt.% is around 10 h. Similarly, the results reported by de Matos et al. [58] showed that the main heat flow peak of a cement paste with a w/c of 0.4 and grey Portland cement without SP occurred approximately at 12 h. The isothermal calorimetry results also indicate that the partial replacement of WC with Nb_2_O_5_ percentages up to 2.0 wt.% does not affect the heat flow and the cumulative heat of the pastes. This suggests that Nb_2_O_5_ did not significantly affect the cement hydration reactions. These findings are consistent with the compressive strength results discussed later. Moreover, it corroborates with previous studies on incorporating other types of nanoparticles in cement-based materials [2,3]. Therefore, it is suggested to evaluate higher Nb_2_O_5_ incorporation contents.

### 3.3. Compressive Strength

**Figure 6:**
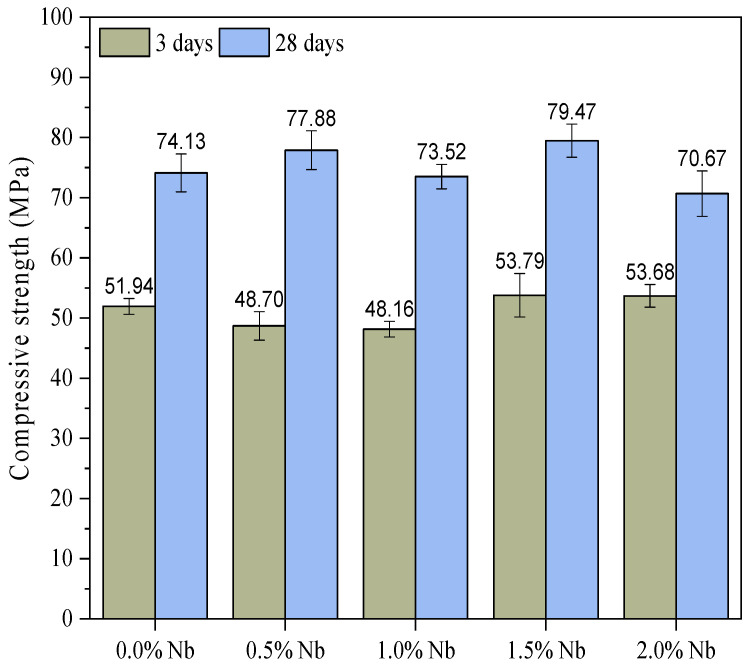
Compressive strength of WC pastes with different Nb_2_O_5_ contents at 3 and 28 days.

**Figure 7:**
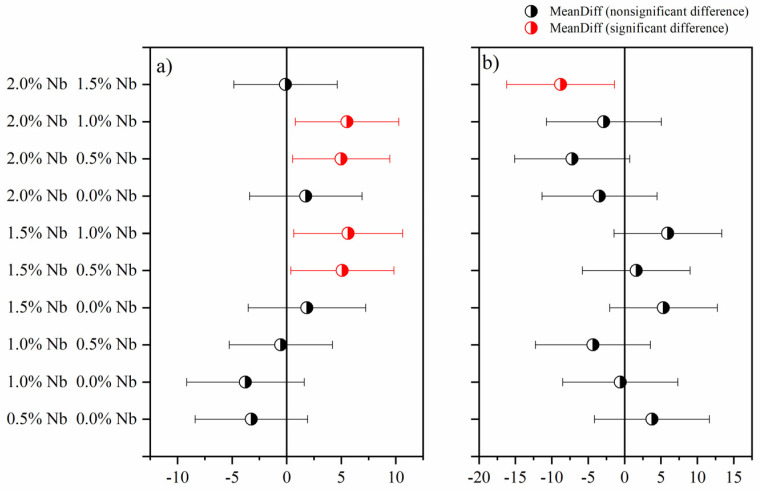
Comparison of average compressive strength at (**a**) 3 and (**b**) 28 days.

The 3 d and 28 d compressive strength values of cement pastes with varying Nb_2_O_5_ contents are presented in Figure 6. The statistical analysis of the compressive strength results is presented in Table 4, indicating that the Nb_2_O_5_ content, age, and their interaction significantly influence the evaluated dependent variable. Additionally, the Tukey test (Figure 7), considering a significance level of 0.05, demonstrated that, in general, different Nb_2_O_5_ contents lead to statistically similar average compressive strengths, particularly after 28 days of hydration. Since there are no prior studies examining the impact of incorporating Nb_2_O_5_ nanoparticles in CBMs, a direct comparison of the results is not possible. Nevertheless, it is likely that the assessed contents (up to 2.0 wt.%) were insufficient to significantly affect the cement hydration reactions, thereby not significantly influencing the porosity and mechanical behavior of the cement pastes. Additionally, another hypothesis pertains to the dispersion of nanomaterials. Insufficient sonication time for nanoparticle dispersion may have also influenced the compressive strength results.

As previously mentioned, no results were found in the literature regarding the influence of Nb_2_O_5_ incorporation on the compressive strength of cementitious matrices. Thus, for comparison purposes, this study’s results were compared with those of studies that evaluated the incorporation of TiO_2_ in cementitious matrices. Meng et al. [59] reported that the incorporation of nano-TiO_2_ on Portland cement mortars increased the early age strength (1 day) and slightly decreased the compressive strength at later ages (e.g., 28 days). For instance, replacement contents of 5.0 and 10.0 wt.% of cement by nano-TiO_2_ increased the 1-d compressive strength by 46.0% and 47.0%, respectively, while decreasing by 6.0% and 9.0% the 28-d compressive strength compared to the control sample. Sobhy et al. [60] evaluated the effect of the addition of TiO_2_ nanoparticles contents of 0.5, 1.0, and 1.5 wt.% on the compressive strength of Portland cement concretes. The results indicated that a TiO_2_ content of 1.5 wt.% increased by 21.53% (7 days), 21.0% (28 days), and 36.57% (91 days) the compressive strength values compared to the plain concrete. According to the authors, these increases can be attributed to the porosity reduction caused by incorporating a nanomaterial since the TiO_2_ is a non-reactive fine filler with no pozzolanic activity. Ren et al. [61] reported that the optimal TiO_2_ content regarding the 28-d compressive strength is 3.0 wt.%, resulting in an increase of 9.22% compared to the control concrete. The authors justify that TiO_2_ improves concrete compressive strength by filling pores and reducing the orientated growth of calcium hydroxide.

### 3.4. Photocatalytic Activity

**Figure 8:**
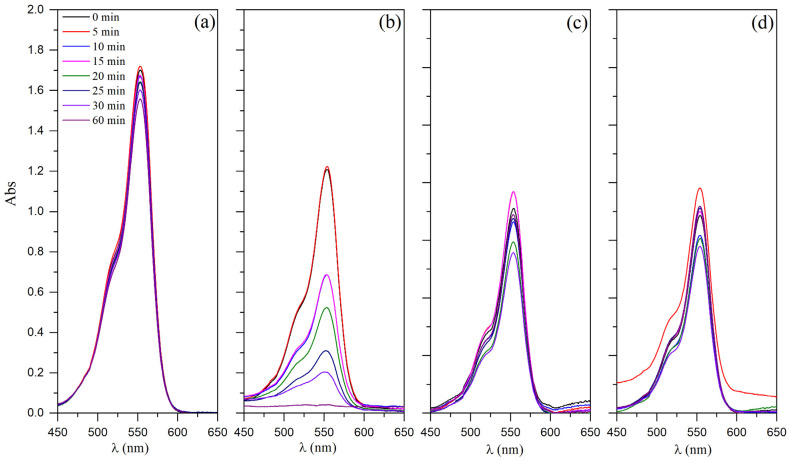
Absorption spectra of RhB solution during irradiation (λ  =  393 nm) (**a**) without photocatalyst, and in the presence of different materials (**b**) commercial Nb_2_O_5_, (**c**) 0.0% Nb cement paste, and (**d**) 2.0% Nb cement paste, at a selected wavelength range.

The photodegradation efficiency of Nb_2_O_5_ and cement pastes (0.0% Nb and 2.0% Nb) were studied by monitoring the UV–Vis absorbance spectra of RhB at a maximum peak of 554 nm, as shown in Figure 8. Overall, all evaluated samples exhibited a reduction in the peak attributed to RhB over time. According to the Lambert–Beer Law, absorbance can be directly related to the concentration of a solution. The greater the absorbance, the greater the concentration of the solution [62]. Therefore, the results demonstrated a decrease in the absorbance peak value associated with RhB, indicating a noticeable loss of color in the solution. Although not shown here (see Figure S1 in the Supplementary Material), the spectrum of the samples also showed a peak near 230 nm, assigned to the hydrogen peroxide used as an oxidizing agent. The photolysis test results (Figure 8a) clearly demonstrate the inefficiency of using H_2_O_2_ and UV light alone, without a catalyst, for the photodegradation of RhB. Nevertheless, previous research has indicated that a more efficient discoloration of RhB can be achieved by combining hydrogen peroxide (Fenton-like process) with a heterogeneous catalyst [63]. Considering the photocatalysis in the presence of H_2_O_2_, commercial Nb_2_O_5_ (Figure 8b) completely degraded RhB within 60 min. On the other hand, both cement samples without (Figure 8c) or with (Figure 8d) Nb_2_O_5_ exhibited inefficient photodegradation of RhB.

Prior to the photocatalytic reaction, a 20-min dark pretreatment was performed to stabilize the adsorption-desorption equilibrium, which resulted in a significant decrease in the solution’s color, indicating an adsorption process. Thus, the initial absorbance discrepancy observed in Figure 8 can be attributed to RhB adsorption. During this period, the solution containing commercial Nb_2_O_5_ exhibited a 30% decrease in color intensity, while the cement sample showed a more significant discoloration of 60%. The literature has reported a similar behavior for cementitious composites with Nb_2_O_5_ [22]. This can be attributed to the difference in pH after adding the catalyst. Niobium oxides possess Brønsted acid and Lewis acid sites on their surface, and when added to RhB solution, its pH decreased from 7 to 4 [64,65]. Conversely, when the cement sample was added, the pH increased from 7 to 10.

It is well known that the pH affects the surface charge on the adsorbent and the adsorbate species in the solution. As previously reported, RhB presented a protolytic equilibrium in water with pKa at 3.22 at 25 °C involving the cationic (*RhB*^+^) and the zwitterionic (*RhB*^±^) forms. It is worth mentioning that the *RhB*^±^ can exist in tautomeric equilibrium with the colorless lactone form (*RhB*^0^). However, in water, this tautomeric equilibrium is shifted towards the RhB species, which is stabilized by the H–bonds [66]. Thus, in an aqueous medium, the equilibrium of *RhB*^+^ ⇌ *RhB*^±^ prevails, and both species present very similar electronic absorption and molar absorptivity [67]. In the present study, pH values range from 4 to 10, with the observed spectra attributed mainly to the *RhB*^±^ species. Therefore, depending on the medium, RhB can interact differently with the catalyst’s surface. In addition, other metallic oxides present in the composition of white Portland cement may have provided more significant dye adsorption.

**Figure 9:**
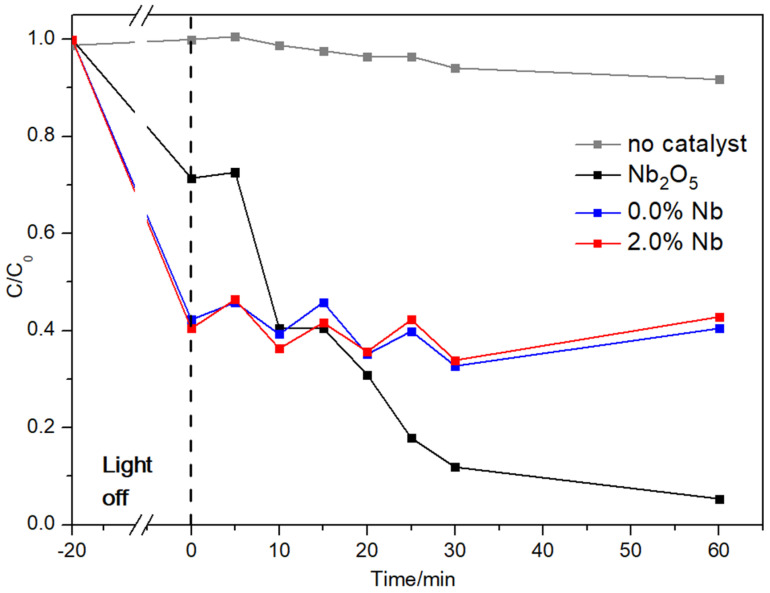
Kinetic curves for discoloration of Rhodamine B dye without catalyst (gray line), using Nb_2_O_5_ commercial (black line), 0.0% Nb cement paste (blue line), and 2.0% Nb cement paste (red line).

The ratio between the final and initial RhB concentrations was calculated (C/C_0_), and the results are shown in Figure 9.

After 20 min in the dark, the concentration of the dye solution was observed to decrease to less than 6 mg L^−1^. To evaluate the efficiency of the photocatalytic tests, the concentration of the solutions was determined by referring to the calibration curve (r^2^ = 0.999) provided in the Supplementary Material (Figure S2). Initially, photolysis was conducted without a catalyst, and the C/C_0_ values remained practically unchanged, indicating a low degradation of RhB due to insufficient energy supplied to break the bonds of RhB dye molecules [22]. While the cement samples showed stabilization of RhB solution discoloration, the commercial Nb_2_O_5_ particles showed a significant photocatalytic activity from 10 min onwards, resulting in a substantial reduction in the C/C_0_ ratio. For instance, the C/C_0_ ratio of Nb_2_O_5_ samples reduced from approximately 0.4 (at 10 min) to 0.05 (at 60 min). On the other hand, the 0.0% Nb and 2.0% Nb cement pastes stabilized the RhB discoloration, with a C/C_0_ ratio close to 0.4 throughout the test. These results suggest that the incorporated contents of Nb_2_O_5_ nanoparticles were insufficient to increase the photodegradation capacity of organic compounds when inserted into cement matrices. This could be due to the low content of nanoparticles or the method of incorporation, which may not have favored the desired effect. Senff et al. [51] also observed that pure TiO_2_ powders showed higher photocatalytic activity than cement pastes containing micro- and nano-TiO_2_ directly incorporated in the mixture.

Moreira et al. [22] assessed the photocatalytic activity of cement mortars containing 2.0 wt.% of Nb_2_O_5_ using methylene blue dye. They observed a reduction in the dye concentration over time for the plain mortar. The results obtained by the authors indicated that the Nb_2_O_5_ incorporation hindered the photocatalytic capacity of mortar. The authors hypothesized that the highly acidic surface groups of Nb_2_O_5_ react with the carbonate groups. The acidic environment favors the release of CO_2_ from carbonates which can reduce the photocatalytic activity of cement-based materials [22].

According to Yousefi et al. [68], the previous dispersion of nano-TiO_2_ in saturated lime water by ultrasound before the incorporation in cement powder favors the formation of CaTiO_3_ through a reaction with Ca(OH)_2_. Moreover, the authors observed that the CaTiO_3_-containing samples enhanced the photocatalytic activity of cement materials under UV radiation and visible light. Thus, it is essential to understand the possible interactions between Nb_2_O_5_ and cement hydration products, as this can directly affect the photocatalytic activity of cementitious materials.

**Figure 10:**
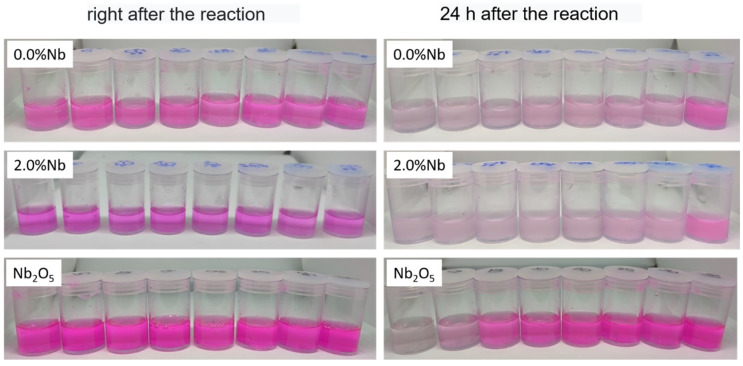
Photographic images of aliquots taken during the 60 min of the Rhodamine B photodegradation reactions using 0.0% Nb, 2.0% Nb and commercial Nb_2_O_5_ right after the reaction and 24 h after the reaction.

The discoloration of RhB dye solution of 0.0% Nb and 2.0% Nb cement pastes was also visually assessed according to the images presented in Figure 10. As previously discussed, the CBM samples showed the concentration of the RhB solution remained stable during the 60 min of photodegradation reaction. However, after 24 h, an interesting discoloration effect was observed in these samples, even if the samples had been filtered after each reaction time. Moreover, no significant differences in the RhB degradation were observed with incorporating a Nb_2_O_5_ content of 2.0 wt.%, which agrees with the previously presented UV–Vis results.

To compare the results obtained with the cement samples, the photodegradation of RhB, using commercial Nb_2_O_5_, was also evaluated at pH 10 solution. The absorption spectra can be seen in Figure S3 in the Supplementary Material. These results showed that discoloration of the RhB solution also occurs. Still, this decrease is less expressive than in the presence of cementitious material, occurring mainly in the first aliquots collected during the photodegradation reaction. Overall, the literature is scarce on the photocatalytic activity of cementitious materials, especially concerning longer analysis periods (24 h).

To better understand this effect, some tests were carried out and allowed to conclude that the bleaching process after 24 h is independent of lighting. In addition, the process is dependent on the presence of H_2_O_2_ in the alkaline medium. Furthermore, it was verified through acidification of the medium ([HCl] = 0.1 mol L^−1^) that the absence of color is not related to the RB^0^ tautomer (colorless). Therefore, the irreversibility of color proves the degradation of RhB. Under these circumstances and based on the work of Verma et al. [69], the discoloration of RhB was associated with the alkaline nature of cement in the presence of H_2_O_2,_ which promotes the formation of superoxide anion radicals. This phenomenon is illustrated in reactions (R1) to (R4):

(R1) $$2{OH}^{-}+H_{2}O_{2}\to O_{2}^{-2}+2H_{2}O$$

(R2) $$O_{2}^{-2}+H_{2}O_{2}\to {2O}_{2}^{-}+2H_{2}O$$

(R3) $$RhB+2O_{2}^{-}+H_{2}O\to intermediate/mineralizedproducts+CO_{2}+2HO^{-}$$

(R4) $$CO_{2}+2O_{2}^{-}\to K_{2}CO_{3}+\frac{3}{2}O_{2}$$

Therefore, a photon-independent process of RhB degradation was observed, which appears to be mediated by the combination of OH^−^/H_2_O_2_ in an aqueous medium. The alkaline properties of cement, along with the presence of H_2_O_2_, constitute an intriguing degradation mechanism that is independent of light. As such, this serendipitous find will be investigated in future studies to understand better the impact of H_2_O_2_ concentration and the presence of different ions.

## 4. Conclusions

This study assessed the effect of different Nb_2_O_5_ contents (0.0, 0.5, 1.0, 1.5, and 2.0 wt.%) on the rheological behavior, hydration kinetics, compressive strength, and photocatalytic activity of white cement pastes. The following conclusions can be drawn:

- The dynamic yield stress and equivalent viscosity of white cement pastes progressively increased over time (0–120 min). This increase is associated with system flocculation and the formation of a rigid network caused by the precipitation of hydration products. The higher SSA of Nb_2_O_5_ nanoparticles contributed to the increase in rheological parameters in cement pastes up to a content of 1.5 wt.%. For a higher content (2.0 wt.%), possibly the Nb_2_O_5_ incorporation slightly delayed the initial hydration reactions of white cement, resulting in viscosity values up to 23.0% lower than the plain cement paste.
- The isothermal calorimetry results indicated that the incorporation of Nb_2_O_5_ up to a content of 2.0 wt.% did not have a significant impact on the induction period, main heat flow peak, and cumulative heat after 100 h of hydration in white cement pastes.
- Compressive strength results also indicated that the Nb_2_O_5_ evaluated contents did not significantly affect white cement paste’s 3-d and 28-d compressive strength. Two possible hypotheses can be suggested: (i) the Nb_2_O_5_ contents evaluated are low to produce a noticeable effect, or (ii) the dispersion of the nanomaterial was not adequate, thereby compromising its ability to reinforce the cement matrix.
- A photocatalytic activity was observed for Nb_2_O_5_ samples, with RhB concentration C/C_0_ = 0.05 after 60 min exposure to UV–Vis light. Cement pastes samples exhibited a C/C_0_ = 0.40 after 60 min. Moreover, the Nb_2_O_5_ content of 2.0 wt.% did not enhance cement materials’ photocatalytic activity suggesting the discoloration of the RhB solution.
- An alternative process of RhB degradation was observed, which did not rely on photon activation but rather on the interaction between OH^−^ from CBM and H_2_O_2_ in an aqueous medium.

Future studies on the topic should focus on evaluating higher percentages of Nb_2_O_5_ replacement in terms of mechanical properties. Additionally, other forms of Nb_2_O_5_ incorporation, such as applying a surface layer in cementitious materials, should be examined to assess the photocatalytic activity of CBMs. Furthermore, it is recommended to investigate the degradation of RhB in contact with cementitious materials.

## Figures and Tables

**Table 1:** Summary of studies on cementitious matrices containing TiO_2_.

Reference	Cementitious Matrix		Dispersion Method		TiO_2_	
	Type	w/c	Type	Duration	Type	Content
[5]	concrete	0.51	high-speed stirrer	5 min	replacement	0.5–2.0%
[6]	paste	0.425	magnetic stirrer	-	replacement	0.5–1.5%
[7]	paste	0.3	sonication	30 min	addition	0.1–1.0%
[24]	paste	0.5	sonication	3 min	replacement	1.0%
[25]	paste	0.3–0.5	-	-	replacement	0.5–2.0%
[10]	mortar	0.64–0.68	-	-	replacement	2.5–10.0%
[26]	concrete	0.38	sonication	-	addition	1.0–3.0%
[27]	paste	-	-	-	replacement	3.0%
[14]	mortar	0.4	sonication	30 min	replacement	1.5–6.0%
[28]	concrete	0.41	-	-	replacement	1.0–5.0%
[29]	paste	0.4	handheld electric mixer	1 min	addition	5.0%
[30]	concrete	0.4	sonication	30 min	replacement	1.0–5.0%
[31]	paste	0.5	sonication	3 min	replacement	1.0%
[32]	mortar	0.45	-	-	addition	1.0–5.0%
[33]	mortar	0.5	sonication	30 min	replacement	1.0–3.0%
[34]	paste	0.45	-	-	addition	1.0–10.0%
[9]	paste	0.35	-	-	addition	5.0–10.0%

**Table 2:** Chemical composition and physical properties of white Portland cement (WC).

	WC
*Chemical composition (%)*	
Al_2_O_3_	2.82
SiO_2_	22.83
Fe_2_O_3_	0.15
CaO	68.48
MgO	0.25
SO_3_	2.37
TiO_2_	0.12
Loss on ignition	2.98
*Physical properties*	
Specific Gravity (g/cm^3^)	3.14
SSA (m^2^/g)	1.34
D10 (µm)	3.30
D50 (µm)	15.48
D90 (µm)	34.48
Average diameter (µm)	16.38

**Table 3:** Detailed composition of cement pastes.

Cement Paste	White Cement (g)	Nb_2_O_5_ (g)	Water (g)	Energy (J)
0.0% Nb	200.00	0.00	80.00	-
0.5% Nb	199.00	1.00	80.00	59,218.00
1.0% Nb	198.00	2.00	80.00	58,314.00
1.5% Nb	197.00	3.00	80.00	57,283.00
2.0% Nb	196.00	4.00	80.00	56,072.00

**Table 4:** ANOVA compressive strength results of cement pastes.

	Degrees of Freedom (DF)	Sum of Squares (SS)	Mean Square (MS)	F Value	*p* Value	Sig ^a^
Nb content	4	139.9263	34.9815	5.1518	0.00324	S
Age	1	5063.0015	5063.0015	745.6459	0.00000	S
Interaction	4	156.3748	39.0937	5.7574	0.00175	S
Error	27	183.3323	6.7900	-	-	-
Total	36	5793.4570	-	-	-	-

^a^ Significance: S—statistically significant.

## Data Availability

Data will be made available on request.

## Supplementary Materials

The following supporting information can be downloaded at: https://www.mdpi.com/article/10.3390/ma16114090/s1, Figure S1: Example of complete absorbance spectra of Rhodamine B solution during the photodegradation reaction. In this example, commercial Nb_2_O_5_ was used as a catalyst in a solution without pH adjustment; Figure S2: Calibration curve of RhB; Figure S3: Absorbance spectra of Rhodamine B solution (pH 10) during the photodegradation reaction with commercial Nb_2_O_5_ as a catalyst.
