# Supplementary material for: Assessing the Rheological, Mechanical, and Photocatalytic Properties of Niobium Oxide-Incorporated White Cement Pastes
Source: Materials (Basel). 2023 May 31;16(11):4090. doi: 10.3390/ma16114090 (PMC10254636; doi:10.3390/ma16114090)
Supplement: Supplementary file 1 [file materials-16-04090-s001.zip › materials-2402172-supplementary.pdf]

Supplementary information for the manuscript

Assessing the Rheological, Mechanical, and Photocatalytic Properties of Niobium Oxide-Incorporated White Cement Pastes

Laura Silvestro[1*], Caroline Maroli[2], Brenda Koch[2], Artur Spat Ruviaro[3], Geannina Lima[3], Mariane Kempka[1], Camila Fabiano de Freitas Marin[2], Daniela Zambelli Mezalira[2] and Philippe Jean Paul Gleize[3]

[1] Coordenação de Engenharia Civil, Universidade Tecnológica Federal do Paraná (UTFPR), Guarapuava, Brasil; laurasilvestro@utfpr.edu.br; marianekempka@professores.utfpr.edu.br
[2] Programa de Pós-Graduação em Química, Universidade Federal de Santa Catarina (UFSC), Brasil; c.maroli@posgrad.ufsc.br; bbrendakoch@gmail.com, camila.f.freitas@ufsc.br, daniela.z.m@ufsc.br
[3] Laboratório de Aplicação de Nanotecnologia em Construção Civil (NANOTEC), Universidade Federal de Santa Catarina (UFSC), Brasil, arturspatruviaro@gmail.com; geanninasantos@hotmail.com; p.gleize@ufsc.br

* Correspondence: laurasilvestro@utfpr.edu.br

This supplementary file contains the following figures:

Figure S1 – Example of complete absorbance spectra of Rhodamine B solution during the photodegradation reaction. In this example, commercial Nb_2O_5 was used as a catalyst in a solution without pH adjustment.
Figure S2 – Calibration curve of RhB.
Figure S3 – Absorbance spectra of Rhodamine B solution (pH 10) during the photodegradation reaction with commercial Nb_2O_5 as a catalyst.

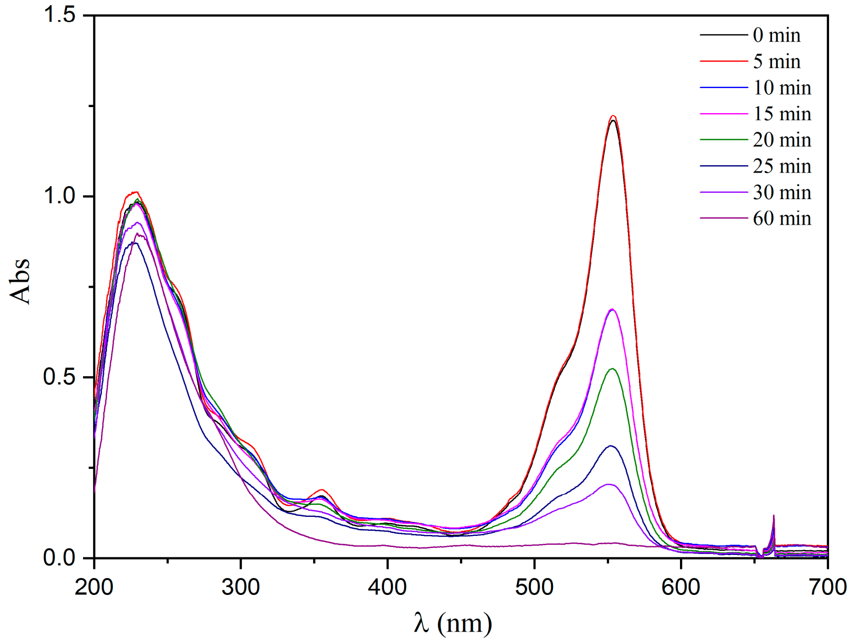

Figure S1. Example of complete absorbance spectra of Rhodamine B solution during the photodegradation reaction. In this example, commercial Nb_2O_5 was used as a catalyst in a solution without pH adjustment.

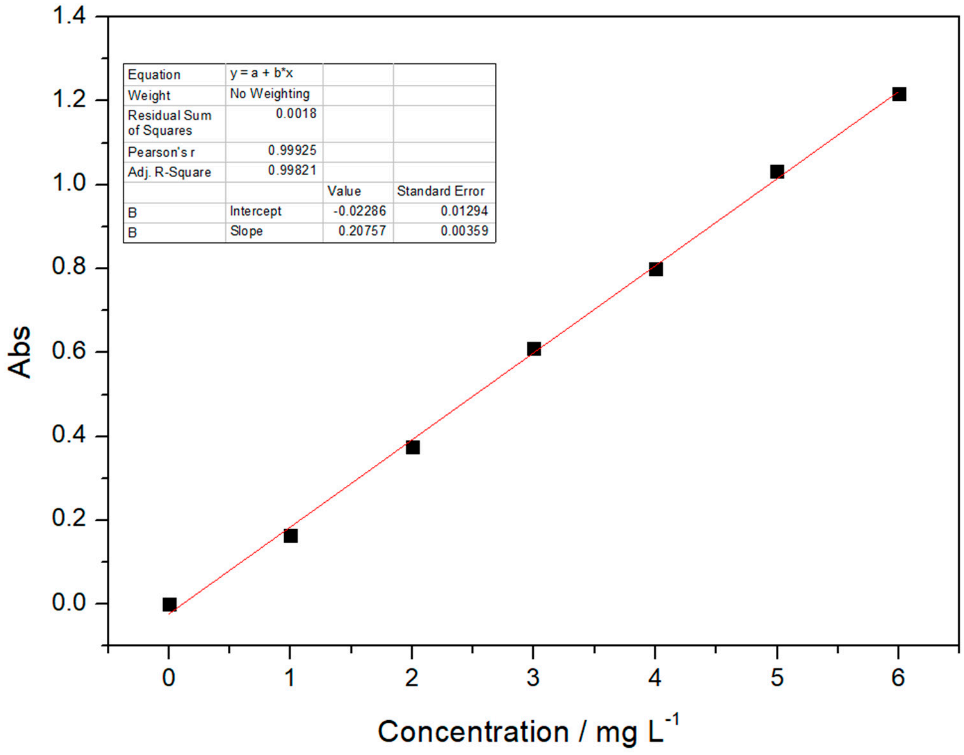

Figure S2. Calibration curve of RhB solution.

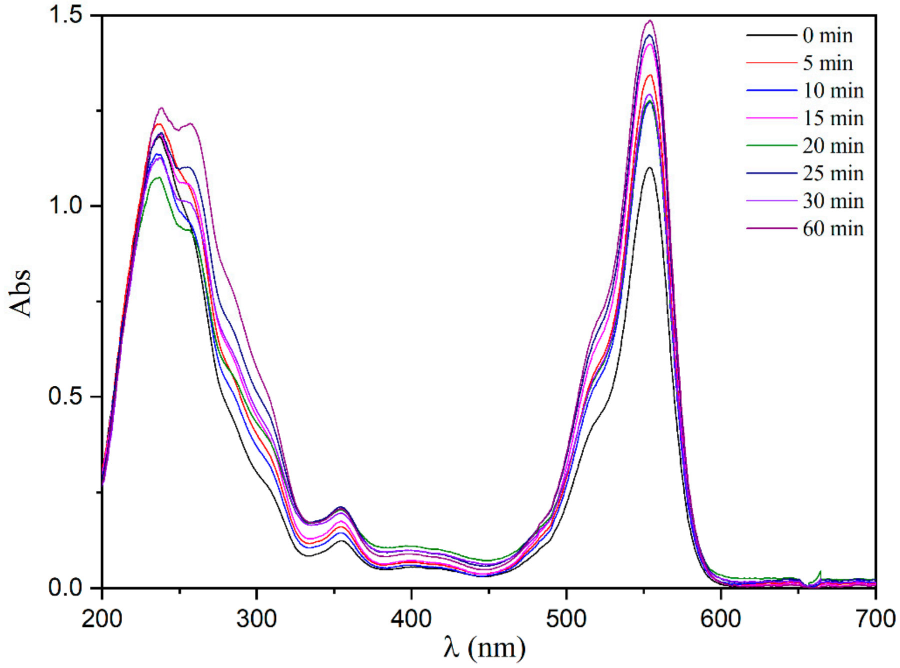

Figure S3. Absorbance spectra of Rhodamine B solution (pH 10) during the photodegradation reaction with commercial Nb_2O_5 as a catalyst.
